# Adolescent sexual and reproductive health in sub-Saharan Africa: who is left behind?

**DOI:** 10.1136/bmjgh-2019-002231

**Published:** 2020-01-26

**Authors:** Dessalegn Y Melesse, Martin K Mutua, Allysha Choudhury, Yohannes D Wado, Cheikh M Faye, Sarah Neal, Ties Boerma

**Affiliations:** 1 Countdown to 2030 for Women's, Children's and Adolescents' Health, Centre for Global Public Health, Department of Community Health Sciences, University of Manitoba, Winnipeg, Manitoba, Canada; 2 African Population and Health Research Center, Nairobi, Kenya; 3 Data and Analytics Section, UNICEF, New York City, New York, USA; 4 West Africa Regional Office, African Population and Health Research Center, Dakar, Senegal; 5 Department of Social Statistics and Demography, University of Southampton, Southampton, Hampshire, UK

**Keywords:** health policy, public Health

## Abstract

Adolescent sexual and reproductive health (ASRH) continues to be a major public health challenge in sub-Saharan Africa where child marriage, adolescent childbearing, HIV transmission and low coverage of modern contraceptives are common in many countries. The evidence is still limited on inequalities in ASRH by gender, education, urban–rural residence and household wealth for many critical areas of sexual initiation, fertility, marriage, HIV, condom use and use of modern contraceptives for family planning. We conducted a review of published literature, a synthesis of national representative Demographic and Health Surveys data for 33 countries in sub-Saharan Africa, and analyses of recent trends of 10 countries with surveys in around 2004, 2010 and 2015. Our analysis demonstrates major inequalities and uneven progress in many key ASRH indicators within sub-Saharan Africa. Gender gaps are large with little evidence of change in gaps in age at sexual debut and first marriage, resulting in adolescent girls remaining particularly vulnerable to poor sexual health outcomes. There are also major and persistent inequalities in ASRH indicators by education, urban–rural residence and economic status of the household which need to be addressed to make progress towards the goal of equity as part of the sustainable development goals and universal health coverage. These persistent inequalities suggest the need for multisectoral approaches, which address the structural issues underlying poor ASRH, such as education, poverty, gender-based violence and lack of economic opportunity.

**Summary box**

- Adolescent sexual and reproductive health remains a major public health issue in sub-Saharan Africa, especially for adolescent girls.
- Even though there has been some overall progress in indicators related to adolescent sexual behaviour, marriage, fertility, family planning and HIV, most gaps by urban–rural residence, level of education and poorest–richest households are persisting and need much greater attention.
- Within sub-Saharan Africa, there are major differences in adolescent sexual and reproductive health between West and Central Africa and Eastern and Southern Africa, and by country within those regions, demonstrating the importance of country-specific data to guide policies and programmes to improve adolescent sexual and reproductive health for all adolescents.
- Adolescent sexual and reproductive health policies and programs need to be country specific, culturally sensitive, multisectoral and inclusive in their contents, as the underlying causes include poverty, lack of education and gender-based violence.

## Introduction

Sub-Saharan Africa will be home to over 250 million adolescents aged 10–19 years by 2020, or 20% of all adolescents globally. This proportion is expected to increase to 24% by 2030.^1^ Adolescent sexual and reproductive health (ASRH) is a major public health issue both from the perspective of the prevention of unintended pregnancies and sexually transmitted infections (STIs) including HIV.^2 3^

Sub-Saharan Africa has made progress in improving ASRH outcomes over the last few decades. The adolescent fertility rate has fallen from 126 per 1000 live births during 2000–2005 to 103 during 2015–2020,^1^ while child marriage, defined as a marriage or a union before the age of 18 years, is also declining.^4^ Contraceptive prevalence among single women 15–24 years has risen from 23% in 1996–2000 to 33% in 2011–2015.^5^ The HIV epidemic among adolescents appears in the region to have at least stalled in most countries. However, these gains are only small and not universal and there is some evidence that not all are benefiting.^6 7^ The Sustainable Development Goals (SDGs) pledge to ‘leave no-one behind’ has ensured that monitoring disparities and acting to reduce inequities has risen on international and national agendas, but until recently the response on ASRH has been subdued.

In this paper, we show that ASRH in sub-Saharan Africa continues to be a major public health challenge with evidence of marked inequity. We consider four key and inter-related dimensions of inequality (gender, education, urban–rural residence and wealth) for analysis, with a focus on six critical areas of ASRH (sexual initiation, marriage, fertility, HIV, condom use and use of modern contraceptives for family planning) which are closely linked to each other. We also discuss data limitations when analysing trends and characteristics.

## Approach

Our analysis is based on a review of published literature (with a focus on multicountry studies within sub-Saharan Africa), as well as the results of our synthesis of national representative Demographic and Health Surveys (DHS) data for 33 countries in sub-Saharan Africa with a most recent survey since 2010 (data extracted from StatCompiler) and national HIV surveys.^6 7^ The surveys were on average conducted in 2014. We computed the median ages at first sex, marriage and birth based on the recall of these events by women and men aged 25–29 years (or 30–34 years if the event median was 25 years or higher).

In order to obtain further insights into more recent trends, we also analysed primary DHS data for respondents 15–24 years in a life table analysis of reproductive events in 10 large-population countries with a survey around 2004, 2010 and 2015. The 10 countries—Ethiopia, Ghana, Kenya, Malawi, Mozambique, Nigeria, Tanzania, Uganda, Zambia and Zimbabwe—are part of the Countdown to 2030 for women’s, children’s and adolescents’ health (http://countdown2030.org/) regional initiative for data analysis in sub-Saharan Africa and were selected because they have populations over 15 million, together accounting for 53% of the total population in sub-Saharan Africa, and conducted three surveys in the relevant periods. The descriptions of the surveys, indicators and methods are shown in online supplementary file 1.

10.1136/bmjgh-2019-002231.supp1 Supplementary data

## Key dimensions of inequality: who is left behind?

### Gender: girls continue to bear the brunt

ASRH inequalities in sub-Saharan Africa for women are rooted within social, cultural and economic spheres that influence their future prospects, decision-making power and autonomy.^8^ Such gender inequalities are critical for ASRH throughout adolescence, even at ages 10–14 years,^9^ and influence an individuals’ ability to realise his/her sexual and reproductive health rights, especially girls.

**Figure 1:**
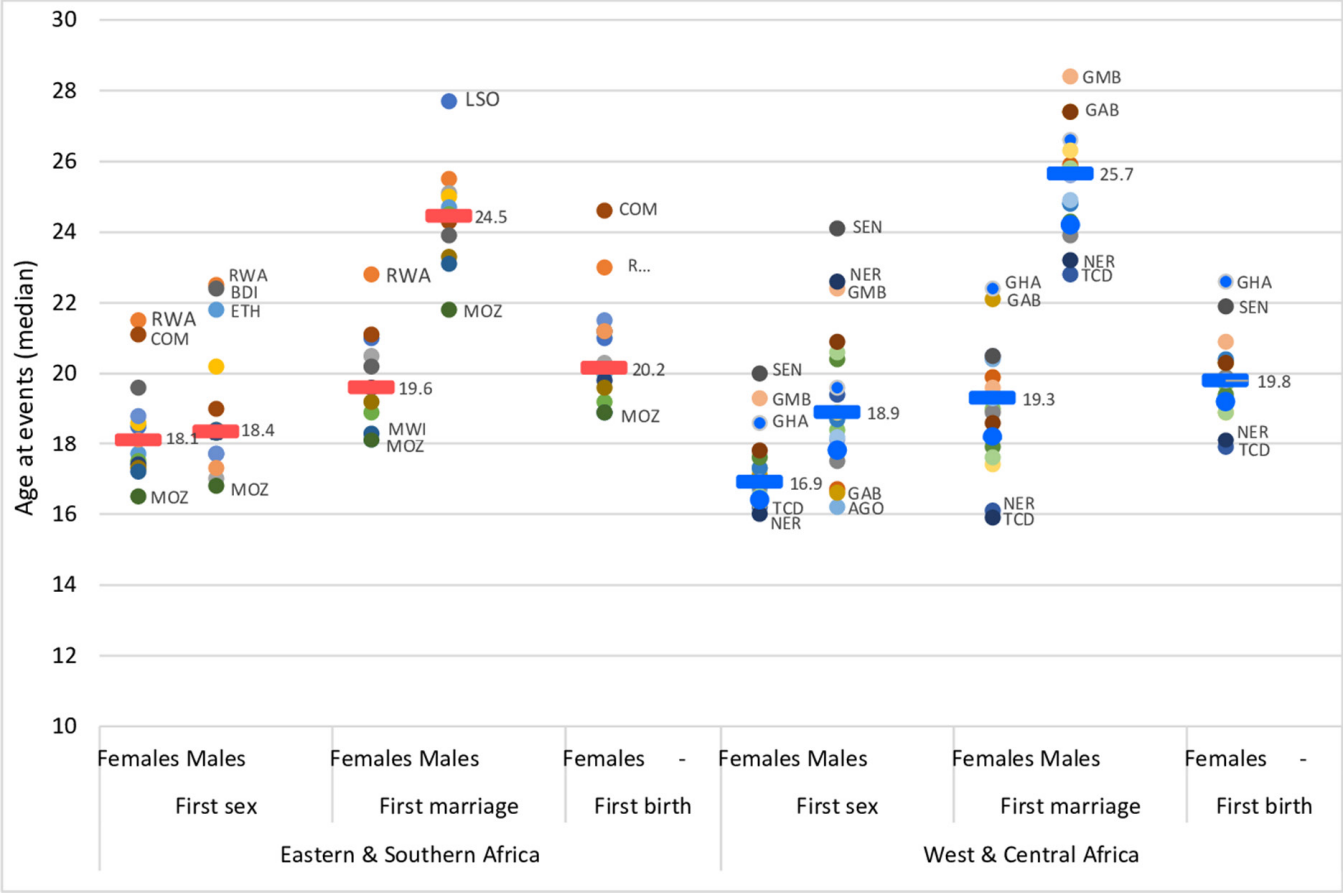
Median age at first sex, age at first marriage and age at first birth in 33 countries in sub-Saharan Africa (14 countries in Eastern and Southern and 19 countries in West and Central Africa), most recent DHS 2010–2018. Dash denotes median value of countries in subregion. Dot denotes country median age at first sex, marriage or birth for both men and women aged 25–29 years (or 30–34 years for men only if the median age at first marriage was 25 years or higher). AGO, Angola; BDI, Burundi; COM, Comoros; ETH, Ethiopia; GMB, Gambia; GAB, Gabon; GHA, Ghana; LSO, Lesotho; MWI, Malawi; MOZ, Mozambique; NER, Niger; RWA, Rwanda; SEN, Senegal; TCD, Chad.

*Sexual initiation*, which occurs at similar age range in sub-Saharan Africa as many other parts of the world,^10^ marks the beginning of exposure to a range of sexual and reproductive health risks such as early pregnancy and HIV infection. In the most recent DHS in 33 countries, the median ages at first sex were 17.4 and 18.4 years for girls and boys, respectively (figure 1 and table 1). There was widespread variation between countries, ranging from 16.0 to 21.5 years for females (Mozambique and Rwanda, respectively) and from 16.2 to 24.1 years for males (Angola and Senegal, respectively). The gender gap was much larger in West and Central Africa (2.0 years) than in Eastern and Southern Africa (0.3 years), mainly due to a younger age at first sex among girls in West and Central Africa. Our trend analysis for 10 countries showed a reduction of the gender gap for age at first sex during 2004–2015. The median age at first sex for girls increased from 17.4 to 17.7 years but a small decrease was observed for boys, reducing the gender gap to 0.2 years in the most recent surveys (table 2).

**Table 1:** Key indicators of adolescent sexual and reproductive health (country medians), 33 countries with most recent DHS since 2010*, sub-Saharan Africa by subregion

	Eastern and Southern Africa		West and Central Africa		Sub-Saharan Africa	
	Girls	Boys	Girls	Boys	Girls	Boys
Median age at first sex (years)	18.1	18.4	16.9	18.9	17.4	18.4
Median age at first marriage (years)^†^	19.6	24.5	19.3	25.7	19.4	24.9
Median age at first birth (years)	20.2	---	19.8	----	19.9	----
Family planning coverage, married women 15–19 years (%)	55.6	---	20.1	---	29.3	----
Family planning coverage, sexually active single women 15–19 years (%)	50.1	---	33.6	---	43.3	---
HIV prevalence, 15–19 years (%)	3.3	1.5	0.8	0.4	1.2	0.5
Condom use at last sex with non-regular partner, 15–19 years (%)	43.2	58.0	31.8	46.2	37.9	52.1

Number of surveys for HIV prevalence obtained from DHS and other national surveys with HIV testing for 32 countries; Family planning coverage is defined as demand for family planning satisfied with modern methods of contraception, taking into account contraceptive prevalence and unmet need; median age at first sex, marriage and birth obtained from men and women aged 25–29.

*Number of countries is number of surveys used to compute summary measure of respective indicators.

†Median age at first marriage among men aged 30–34 was used to impute if median exceeded 25 years.

DHS, Demographic and Health Survey.

**Table 2:** Trends in age at first sex, age at first marriage and age at first birth in years, median value from 10 countries, 2004–2015

			Females			Males			Male–female gap*		
			2004	2010	2015	2004	2010	2015	2004	2010	2015
Age at first sex		All	17.4	17.7	17.7	18.1	18.2	17.9	0.7	0.5	0.2
	Residence	Rural	17.2	17.3	17.3	18.1	18.3	17.9	0.9	1.0	0.6
		Urban	18.1	18.4	18.5	18.2	18.5	18.6	0.1	0.1	0.1
		Gap	0.9	1.0	1.1	0.2	0.2	0.8			
	Education level	None	16.3	16.2	16.1	19	18.3	18.4	2.7	2.1	2.3
		Primary	17.2	17.1	16.9	17.9	18.0	17.6	0.7	0.9	0.7
		Secondary+	18.8	18.9	18.7	18.7	18.8	18.4	−0.1	−0.1	−0.3
		Gap†	2.5	2.7	2.6	−0.3	0.5	0.1			
	Wealth tertile	Poorest	16.8	17.1	17	18.2	18	17.8	1.4	0.9	0.8
		Middle	17.3	17.7	17.7	18.1	18.5	17.7	0.8	0.8	0
		Wealthiest	18.2	18.6	18.8	18.2	18.6	18.6	0	0	−0.2
		Gap‡	1.4	1.5	1.8	0	0.5	0.8			
Age at first marriage		All	19.0	19.7	19.7	24.3	24.6	25.1	5.3	4.9	5.4
	Residence	Rural	18.4	18.7	18.8	23.8	23.6	23.9	5.4	4.9	5.1
		Urban	21.1	21.5	21.8	25.6	26.3	26.1	4.5	4.8	4.3
		Gap	2.7	2.8	3	1.8	2.7	2.2			
	Education level	None	17.2	17	17.1	22.7	22.5	23.2	5.5	5.5	6.1
		Primary	18.5	18.3	18.3	24.3	24.7	23.9	5.8	6.4	5.6
		Secondary+	22.8	21.8	22.7	25.1	25.8	25.4	2.3	4.0	2.7
		Gap†	5.6	4.7	5.6	2.5	3.3	2.2			
	Wealth tertile	Poorest	18	17.9	18.1	23.0	23.0	23.3	5.0	5.1	5.2
		Middle	18.4	18.8	19.3	23.8	24.1	24.5	5.4	5.3	5.2
		Wealthiest	21.2	21.6	22.3	25.7	26.6	26.2	4.5	5.0	3.9
		Gap‡	3.2	3.7	4.2	2.7	3.6	2.9			
Age at first birth		All	20.0	20.0	20.1						
	Residence	Rural	19.5	19.5	19.3						
		Urban	20.8	21.8	21.6						
		Gap	1.3	2.3	2.3						
	Education level	None	18.5	18.2	18.2						
		Primary	19.1	19.1	19						
		Secondary+	22.8	22.8	22						
		Gap†	4.4	4.6	3.8						
	Wealth tertile	Poorest	19	18.9	18.9						
		Middle	20.1	19.7	19.7						
		Wealthiest	21.2	21.8	22.6						
		Gap‡	2.3	2.9	3.7						

Data used for analysis were from all adolescents and young women and men aged 15–24, except age at first marriage for men where data is from all men aged 15–29.

*Absolute difference in median between male and female adolescents.

†Absolute difference in median age between secondary+ (i.e., secondary or higher education level) and none (refers to those with no education).

‡Absolute difference in median age between wealthiest and poorest wealth tercile.

Marriage patterns in sub-Saharan Africa are characterised by substantial age differences between the partners and high prevalence of child marriage among girls (before age 18).^11^ Child marriage, which is an SDG indicator, is not only a violation of a young person’s rights with potentially far-reaching consequences for ASRH and educational opportunities.^12^ While child marriage does occur among boys, in most countries it is far less common than among girls.^13^ In our analysis of the 33 countries the median ages at first marriage among women 25–29 and men 25–34 years were 19.4 and 24.9 years, respectively (figure 1 and table 1). The median male–female age at marriage gaps were 4.9 and 6.4 years in Eastern and Southern and West and Central Africa, respectively. Several studies have reported an increase in age at first marriage and decline in child marriage during the last decades.^14 15^ The reductions in child marriage, however, were not uniform, with several countries showing no change over time such as Chad and Niger where over 60% of all girls were married before the age of 18 years.^14^ In our analysis of 10 countries with recent data, age at first marriage increased by 0.7 years for both women and men during 2004–2015 (table 2).

The incidence and prevalence of HIV and other STIs among adolescents remains a key public health challenge in much of sub-Saharan Africa.^16 17^ There is a clear gender divide, with adolescent girls at much higher risk for HIV transmission than boys.^16 18 19^ Sexual transmission plays a key role, as paediatric infections (mother-to-child transmission during the pregnancy, delivery or post partum) are unlikely to cause such gender disparities. In addition to biological factors,^20^ the age difference between sexual partners is an important driver of the epidemic among adolescent girls and young women, because older male partners are more likely to be HIV infected than adolescent boys. Condom use may also be due to limited sexual negotiation power and skills of girls.^21^ In the most recent surveys in 30 countries with HIV testing, the country median HIV prevalence rate among girls 15–19 years was 1.2%, two times higher than for boys of the same age. There are also marked country and regional differences with HIV prevalence at 15–19 years Eastern and Southern Africa four times higher than in West and Central Africa (table 1).

Condom use for HIV/STI prevention is underpinned by complex gender and cultural dynamics within sexual relationships and is driven by the ability for both partners to openly communicate and negotiate protection options.^22^ In 32 countries with data from a recent survey in sub-Saharan Africa, nearly 38% of sexually active adolescent girls reported condom use at last sex with a non-marital non-cohabiting partner, compared with 51% of adolescent boys (table 1). Condom use among both adolescent boys and girls was considerably higher in Eastern and Southern Africa than in West and Central Africa, perhaps because of greater condom promotion efforts due to the more severe HIV epidemics.^5^

## More education: later sex, marriage and childbearing

**Figure 2:**
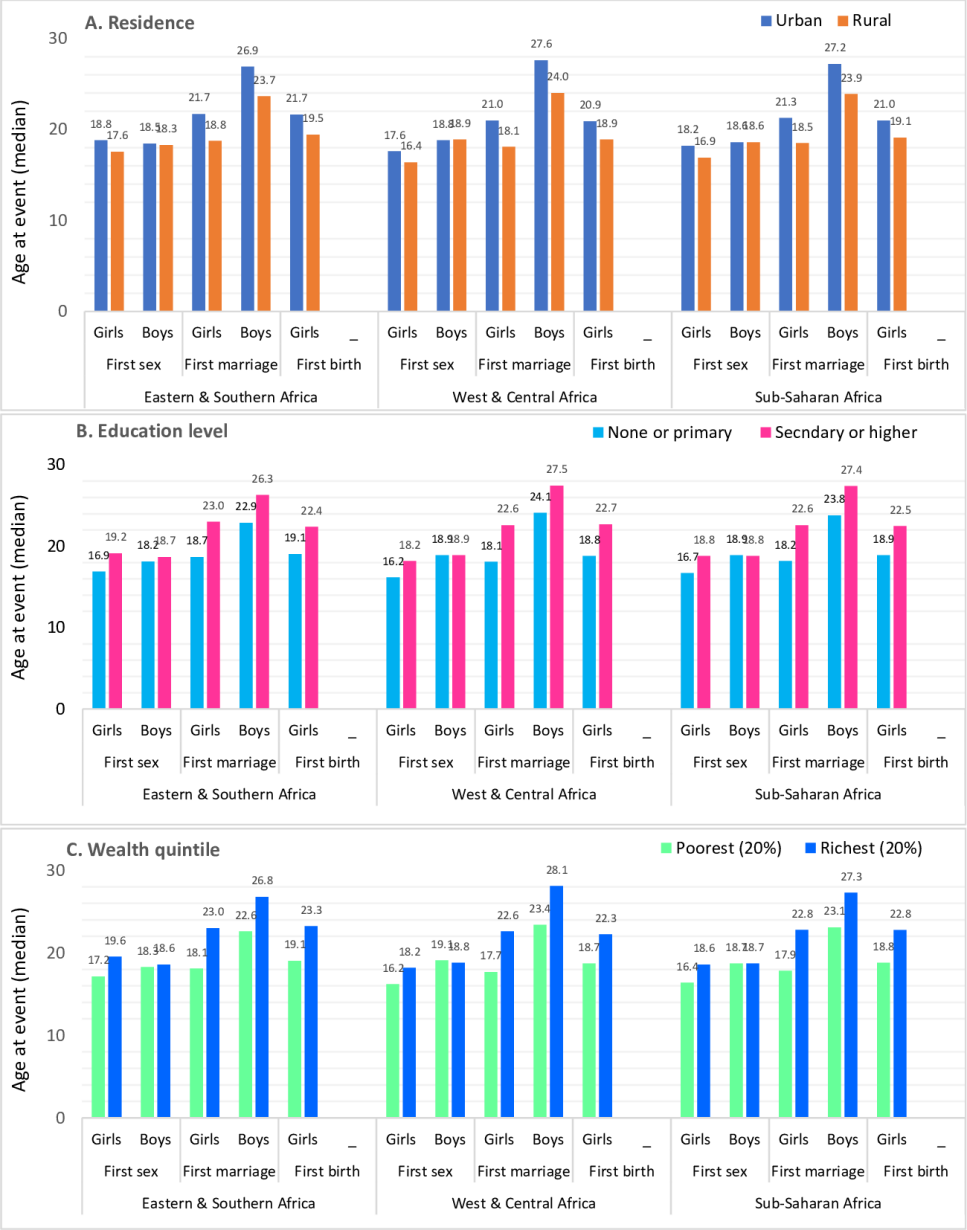
Median age at first sex, age at first marriage and age at first birth for girls and boys, by level of education, urban–rural residence and household wealth, 33 countries in sub-Saharan Africa (14 countries in Eastern and Southern and 19 countries in West and Central Africa), most recent DHS 2010–2018. Data extracted from STATcompiler are available and reported by wealth quintile. DHS, Demographic and Health Survey.

Educational attainment is strongly related to sexual and reproductive behaviours. Higher levels of school attainment are associated with later initiation of sex, marriage and childbirth (figure 2). Girls with less education (none or primary) initiated sex 2.2 years earlier, were married 4.4 years earlier and had their first child 2.5 years earlier than girls with secondary or higher education, based on 33 countries with recent data. Differences in sexual initiation were non-existent for boys, but more educated young men married 3.6 years later than those who were less educated. The gender gaps in age at first sex and marriage by educational status did not reduce much in the 10 countries with surveys around 2004 and 2015 (table 2). Yet, evidence shows that educational changes are an important driver of the overall increases in age at first sex and marriage.^15^ Decomposition analysis showed that this is primarily due to relatively more adolescent girls and boys reaching higher levels of education where later sex and marriage are more common, rather than changes within the educational groups.^23^

Even though there has been an increase in contraceptive use among young single women in sub-Saharan Africa during the last two decades,^5^ family planning coverage (defined as demand satisfied for family planning with modern methods) among adolescent single girls was still only 38% in 32 recent surveys, with considerably higher levels in Eastern and Southern Africa compared with West and Central Africa. For married adolescents, family planning coverage was even lower (table 1). Attending school is associated with greater family planning coverage, but our analysis of trends in nine countries (no recent data for Mozambique) showed that the gap between those with lower and those with higher education reduced in the last decade.

**Figure 3:**
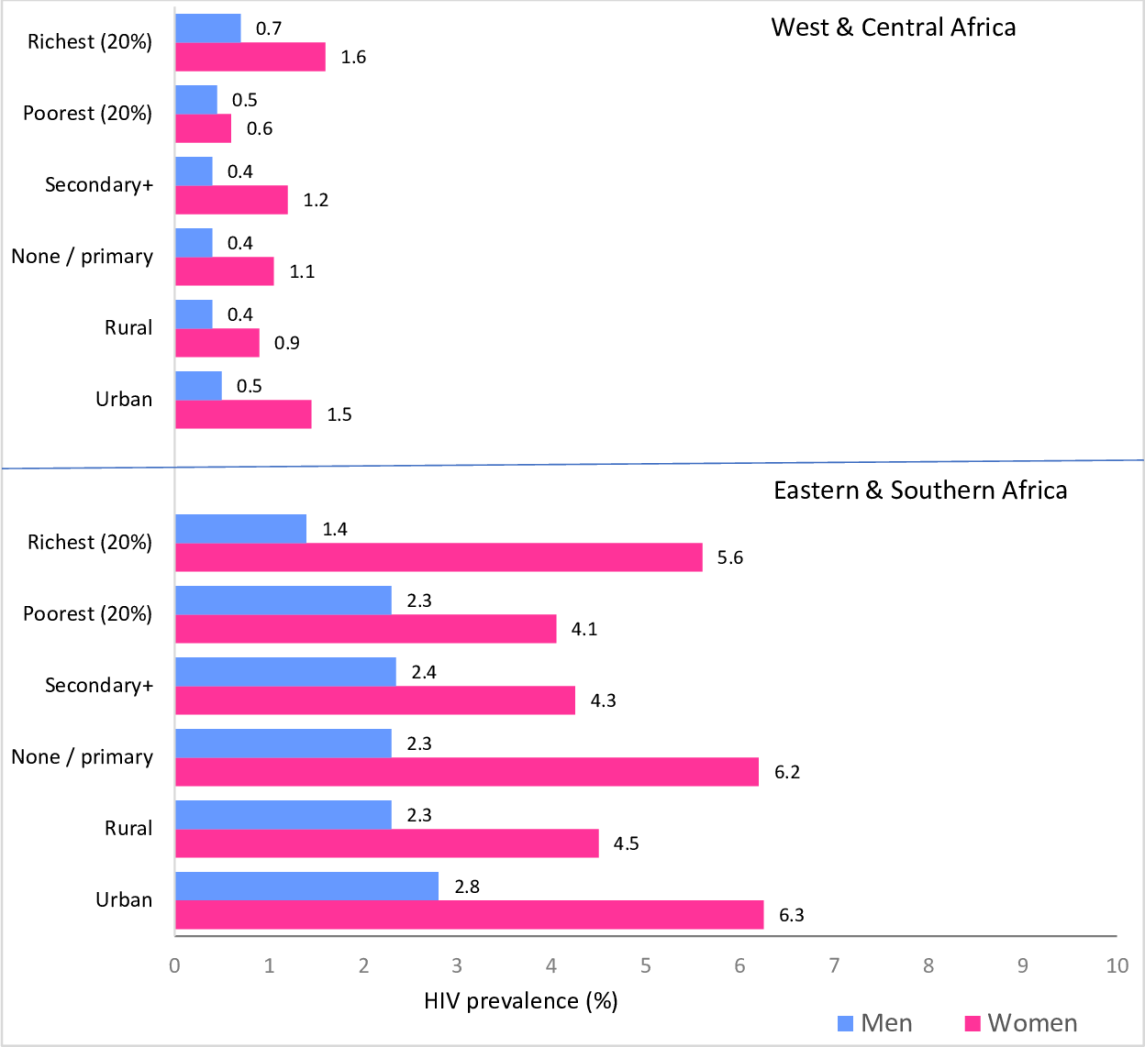
HIV prevalence (%) among girls/women and boys/men 15–24 years, by residence, education level and wealth quintile (poorest vs richest), 28 countries in sub-Saharan Africa (12 countries in Eastern and Southern and 16 countries in West and Central Africa), most recent surveys 2010–2018. Online supplementary file: study populations and data sources, definitions, online supplementary tables 1–8 and online supplementary figures 1–3. Wealth quintile is used as a stratified for consistency and comparability as HIV prevalence data extracted from reports and population-based HIV impact assessemnt (PHIA) are available and reported by wealth quintile.

In the initial years of the HIV epidemic, education was positively associated with HIV prevalence.^24^ Over time, education has become less important as HIV risk factor,^25^ presumably due to greater use of preventive measures (such as sexual partner reduction and condom use) by more educated women and men. A study of trends among people 15–24 years in seven African countries, however, did not find a consistent pattern or change in HIV prevalence by education during 2004–2011.^24^ Our analysis shows that the median HIV prevalence in 30 countries with surveys since 2010 was about the same among both sexes with secondary education or higher compared with those with less education in both subregions of sub-Saharan Africa (figure 3). In Eastern and Southern Africa, HIV prevalence was higher among more educated young women.­

## Urban–rural gap remains wide

A rapidly increasing proportion of adolescents are living in urban settings. According to data from the UN Population Division,^26^ the proportion of adolescents 10–19 years who were living in urban areas in sub-Saharan Africa reached at 38% in 2015 (28% in Eastern and Southern Africa and 48% in West and Central Africa), an increase from nearly 34% in 2005. Adolescents living in urban settings, especially those in low-income settings in metropolitan areas, are more likely to be exposed to greater sexual mixing and risks of HIV than their rural counterparts. At the same time, urban settings may offer better access to contraceptives including condoms, safe abortion and different social norms regarding marriage and fertility. Several studies have shown that adolescents living in urban areas have lower rates of adolescent pregnancy and early marriage, as well as far greater contraceptive use.^27–29^ Most analyses describe urban–rural differences as a dichotomous stratification, but recent analysis suggests that the urban poor often have equally poor indicators to their rural counterparts.^30^ Aggregated analysis of DHS data from 33 countries showed that rural girls started sex 1.3 years earlier and married 2.7 years earlier than urban girls (figure 2). There was no difference for boys in age at first sex, but rural men married 3.3 years earlier than urban men. Rural girls had their first birth almost 2 years earlier than urban girls. The urban–rural gaps in ASRH indicators have not reduced during the past decade, according to our in-depth analysis of 10 countries (table 2). In fact, while median ages at first sex, marriage and birth were increasing, the gaps between the urban and rural adolescents increased during 2004–2015 for both sexes. First births to women under the age of 20 years are becoming increasingly concentrated within rural populations.^31^

The coverage of family planning with modern methods differs greatly between urban and rural adolescents, but data from our 10 country analysis suggests that rural contraceptive use is increasingly faster than among urban populations. Family planning coverage among single sexually active girls 15–24 years increased from 34% to 49% during 2004–2015, reducing the gap with urban girls to almost none (51% in 2004 and 53% in 2015) (table 3). Among married adolescents and young women, there were large increases in family planning coverage during 2004–2015 but the absolute urban–rural gap remained the same (online supplementary figures 1–3).

**Table 3:** Trends in family planning coverage with modern methods (%) by marital status, median values from 10 countries in sub-Saharan Africa, DHS 2004–2015

		Married			Single		
		2004	2010	2015	2004	2010	2015
Residence	Urban	49.3	57.3	73.2	51.0	53.6	52.7
	Rural	29.8	40.1	55.7	33.7	42.7	48.6
Education level	Primary or lower	30.9	42.7	59.1	31.1	40.1	46.0
	Secondary or higher	51.6	61.1	68.7	47.9	51.3	52.3
Wealth tertile	Poorest	21.7	32.8	48.5	27.4	29.7	41.0
	Wealthiest	46.4	58.0	73.7	54.2	55.2	56.0

The 10 countries are Ethiopia, Ghana, Kenya, Malawi, Mozambique, Nigeria, Tanzania, Uganda, Zambia and Zimbabwe. Family planning coverage is defined as demand for family planning satisfied with modern methods of contraception, taking into account contraceptive prevalence and unmet need.

In Eastern and Southern Africa, HIV prevalence is higher in urban than in rural areas, especially among women 15–24 years (medians 6.3% and 4.5%, respectively) (figure 3). In West and Central Africa, urban women also had a higher prevalence than their rural counterparts (medians 1.5% and 0.9%, respectively). It has also been shown that the urban poor have a greater risk of HIV than other urban residents.^32^ Condom use at last sex with a non-marital non-cohabiting partner was 49% and 61% among urban adolescent girls and boys, respectively, which was about 1.5 times higher than among rural adolescent girls.

## Wealth-related inequalities

While there are relatively few multicountry studies examining socioeconomic inequalities in ASRH in sub-Saharan Africa, there is clear evidence of a wealth gradient for a number of indicators such as early marriage, age at first birth and contraceptive usage indicating that poorer adolescents face significant barriers in realising their reproductive health and rights.^33^ The data from the DHS surveys from 33 countries provide ample evidence of large disparities in ASRH by wealth quintile (figure 2). Compared with adolescent girls from the richest wealth quintile, adolescent girls from the poorest wealth quintile had their first sex 2.2 years earlier, married 4.6 years earlier and gave birth 4.1 years earlier (table 2). Boys in both wealth quintiles started sex at the same age, but those in the poorest households married 4.3 years earlier than those in the richest households. HIV prevalence was higher among both sexes in the richest quintiles among 15–24 years in both regions and most countries. Overall, the wealth-related gaps are even larger than for education and place of residence.

For the analyses of trends over time in the 10 countries, we computed wealth tertiles from an asset index score^34^ instead of the more conventional quintiles to reduce sampling errors. The results show that gaps for first sex, marriage and childbearing between the poorest and richest tertiles increased except for marriage among males (table 2). The median age at first birth has increased by 0.8 and 1.3 years among those from urban and wealthiest families, respectively, during the period 2004–2015.

## Conclusion

Our analysis clearly demonstrates major inequalities and uneven progress in many key ASRH indicators within sub-Saharan Africa. Gender gaps are large with little evidence of change in gaps in age at sexual debut and first marriage, resulting in adolescent girls remaining particularly vulnerable to poor sexual health outcomes. There are also major and persistent inequalities in ASRH indicators by education, urban–rural residence and economic status of the household which need to be addressed to make progress towards the goal of equity as part of the SDGs and universal health coverage. These persistent inequalities suggest the need for multisectoral approaches which address the structural issues underlying poor ASRH such as education, poverty, gender-based violence and lack of economic opportunity.^35^

There are marked differences between the two subregions of sub-Saharan Africa used in our analyses, with West and Central Africa lagging behind in adolescent reproductive health, while Eastern and Southern Africa suffers from a more severe HIV epidemic among adolescents. Adolescent girls in West and Central Africa are likely to initiate sex, get married and give birth earlier and have lower family planning coverage, compared with those in Eastern and Southern Africa. Among boys, sexual initiation and marriage are generally later in West and Central Africa compared with similar age group of boys in Eastern and Southern Africa, resulting in a longer period of premarital sex in West and Central Africa. Even though it is possible to identify general regional patterns, there are major country differences within the subregions which deserve further study and have implications for efforts to reduce inequalities in ASRH.

Our data analyses, as well as many of the reviewed publications, relied on national surveys with a particular focus on DHS. This limited the choice of the ASRH indicators and the dimensions of inequality. Several areas were not addressed such as sexual competency, access to comprehensive sexuality education, sexual and gender-based violence and unsafe abortion, for which comparable quality data are still lacking. In addition, while the SDGs are calling for a wide range of equity stratifiers, comparable and reliable data are not available by disability, minorities, religion and migratory status.^36^ New ways of collecting timely and robust data need to be developed and rolled out, including strengthening of health information systems to obtain disaggregated data on access to and use of SRH services by age.

It is also important to recognise that inequalities are likely to be intersectional, which describes the complex way in which characteristics such as gender, poverty, ethnicity, lack of education and rural residence combine and overlap to compound the disadvantage experienced by vulnerable populations. The issue of intersectionality has not been really addressed in relation to ASRH inequality and presents an important research gap. Multivariate analytical methods can play a part in understanding the differing contribution of these characteristics but will be difficult to interpret across different countries and contexts. High-resolution geospatial analysis and modelling drawing on multiple population characteristics within a small area can also be a valuable tool in visualising multiple disadvantages and inequalities reflected within geographic ‘pockets’, and can be used by policy-makers for targeting resources.^37^

Our analysis of survey data is affected by the retrospective nature of the data collected which may lead to recall and social desirability biases in reporting of ASRH events which affect levels and trends. Multiple studies have shown the biases in the reporting of age at first sex and with under-reporting by girls and over-reporting by boys, meaning gender differences in age at first sex may actually be greater than reported.^38–41^ Overstatement of age is also an issue for adolescent reporting of first birth and marriage.^42^ Such biases may become more severe if there is strong emphasis on postponement of first sex, for instance, in the context of HIV control efforts or early marriage is illegal.

Measures of wealth and place of residence are taken at time of reporting, which may be some years after the event of interest. This means that it is impossible to suggest causation, for instance, adolescent pregnancy may either be driven by poverty or cause it, with similar potential relationships with place of residence. There is a lack of longitudinal data within sub-Saharan Africa which would enable analysis of trajectories for young people both before and after key sexual health events, but this would be valuable in further understanding how sexual health outcomes are both influenced by, and, in turn, influence socioeconomic status.

The 2030 SDG targets related to sexual and reproductive health for all adolescents—with health interventions—are still far off in many countries in sub-Saharan Africa. Inequalities in ASRH are still a neglected topic. Our analyses show the persistence of gender, socioeconomic and urban—rural inequalities which needs major attention in ASRH policies and programme. Such policies and programmes need to be country specific, culturally sensitive, multisectoral and inclusive in their contents, as the underlying causes include poverty, lack of education and gender-based violence.
