# Supplementary material for: Adolescent sexual and reproductive health in sub-Saharan Africa: who is left behind?
Source: BMJ Glob Health. 2020 Jan 26;5(1):e002231. doi: 10.1136/bmjgh-2019-002231 (PMC7042602; doi:10.1136/bmjgh-2019-002231)
Supplement: Supplementary data [file bmjgh-2019-002231supp001.pdf]

Supplement

Study populations and data sources

Retrospective data from 33 countries among females and males aged 25-29 years (and 30-34 years for men on age at first marriage) was conducted to better understand the key life events – age at first sex, marriage, and birth – occurred during adolescent age between 15-24. These data were drawn from national representative household surveys, mainly from standard Demographic and Health Surveys (DHS), AIDS Indicator Surveys (AIS), and population-based HIV impact assessment (PHIA).[1,2] For gain further insight into trends, data from 10 countries among adolescent and young female and males age 15-24 were also analysed. These datasets have been utilized as major source of data to produce performance and measurement indicators for low-income and middle-income countries since early 1980s. In this study, only datasets collected from year 2000 onwards were used for analyses.

Indicators definition:

Median age at first sex (years) – age by which 50% of a population has experienced their first sexual intercourse. The indicator was computed from cumulated single-year percent distribution of age reported by woman or man as age in completed years or when they first had sex.[1,3,4] The population here refers to female and male population of age between 15-24 or 25-29 years.

Median age at first marriage (years) – an interpolated calculation of age by which 50% of a population has experienced their first union.[1,3,4] The population here refers to female and male populations of age between 15-24, 25-29 or 30-34 (only for men) years.

Median age at first birth (years) – an interpolated calculation of age by which 50% of a female population has experienced their first live birth.[1,3,4] The population here refers to female population of age between 15-24 or 25-29 years.

HIV prevalence (%) – percentage of young female and male population of particular age group, identified as age between 15-19, 20-24 or combined (15-24), who are HIV infected among respondents who received the test.

Condom use (%) – percentage of young female and male population of particular age group who reported using condom at last sexual intercourse, of all young women or men had sex with more than one partner in the 12 months preceding the survey. The age group refers those between 15-19, 20-24 or combined (15-24).

Melesse DY, *et al. BMJ Global Health* 2020; 5:e002231. doi: 10.1136/bmjgh-2019-002231

Modern contraceptive use for family planning (%) – percentage of modern contraceptive use was computed as the number of sexually active adolescent and young female population of age 15-24 years (identified as married or single) using any modern contraceptive method (implants, IUD, injectable, pills, emergency contraception, condom, sterilization, other) as the numerator divided by the total number of sexually active adolescent and young female population of age between 15-24 (identified as married or single) with a need of family planning (the denominator). Methods with less than 1% were grouped together in the 'others' category.

Results presented in this study were drawn from two main components of analyses: synthesis of data from 33 countries extracted from STATcompiler[4] and analysis of raw data from 10 countries to further triangulate results and better understand who is left behind in adolescents sexual and reproductive health (ASRH) in sub-Saharan Africa.

A. sub-Saharan Africa – synthesis of data from 33 countries

This first component of the analysis involves critical review of published literature and synthesis of data from DHS and AIS from a total of 33 countries in sub-Saharan Africa, using the most recent survey since 2010 and national HIV surveys from a total of 30 countries. The surveys were on average conducted in 2014. The DHS provides standardized nationally representative data that can be disaggregated by major stratifiers, mainly by age group (15-19 vs. 20-24), urban-rural residence, education level (no education, primary vs. secondary or higher), wealth quintile (poorest (20%) vs. richest (20%)). In STATcompiler, data on the median age at first sex, marriage and birth are available for men and women aged 25 or above. Therefore, data on these events were based on the recall of these events by women and men aged 25-29 years, and in particular, data on age at first marriage from men aged 30-34 were used to impute if the data from 25-29 were missing.

HIV prevalence data were extracted from either DHS, AIS or PHIA. HIV prevalence data which were extracted from both STATcompiler and PHIA were used to synthesise inequality. It is worth noting that DHS, AIS and PHIA are all cross-sectional, household, and nationally representative surveys of adults age 15 years and above.

Melesse DY, *et al. BMJ Global Health* 2020; 5:e002231. doi: 10.1136/bmjgh-2019-002231

Supplement Table 1. Data sources utilized for synthesis of key ASRH indicators in sub-Saharan, 33 countries, most recent survey since 2010

Region	Country	Year | Survey	Age at first life events*	Family planning**	condom use***	HIV prevalence
Eastern & Southern Africa	Burundi	2016-17 DHS	x	x	x	x
	Comoros	2012 DHS	x	x	x	
	Ethiopia	2016 DHS	x	x	x	
	Ethiopia	PHIA 2018				x
	Kenya	2014 DHS	x	x	x	
	Kenya	2012 KAIS				x
	Malawi	2015-16 DHS	x	x	x	x
	Mozambique	2015 AIS	x	x	x	x
	Mozambique	2018 MIS				
	Rwanda	2014-15 DHS	x	x	x	x
	Tanzania	2015-16 DHS	x	x	x	
	Tanzania	PHIA 2016				x
	Uganda	2016 DHS	x	x	x	
	Uganda	PHIA 2016				x
	Zambia	2013-14 DHS	x	x	x	
	Zambia	PHIA 2016				x
	Zimbabwe	2015 DHS	x	x	x	
	Zimbabwe	PHIA 2015				x
	Eswatini	PHIA 2016				x
	Lesotho	2014 DHS	X	x	x	
	Lesotho	PHIA 2016				x
	Namibia	2013 DHS	X	x	x	x
	South Africa	2016 DHS	X	x	x	x
West & Central Africa	Angola	2015-16 DHS	X	x	x	x
	Cameroon	2011 DHS	X	x	x	
	Cameroon	PHIA 2017				x
	Chad	2014-15 DHS	x	x	x	x
	Congo	2011-12 DHS	x	x	x	
	Congo Dem.	2013-14 DHS	x	x	x	x
	Gabon	2012 DHS	x	x	x	x
	Benin	2017-18 DHS	x	x	x	
	Burkina Faso	2010 DHS	x	x	x	x
	Cote d'Ivoire	2011-12 DHS	x	x	x	
	Cote d'Ivoire	PHIA 2018				x
	Gambia	2013 DHS	x	x	x	x
	Ghana	2014 DHS	x	x	x	x
	Guinea	2012 DHS	x	x	x	x
	Liberia	2013 DHS	x	x	x	x
	Mali	2018 DHS	x	x	x	x
	Niger	2012 DHS	x		x	x
	Nigeria	2013 DHS	x	x	x	
	Senegal	2017 DHS	x	x	x	x
	Sierra Leone	2013 DHS	x	x	x	x
	Togo	2013-14 DHS	x	x	x	
**Total number of countries**			**33**	**32**	**33**	**30**

*Age at events refers to age at first sex, marriage and birth. **Family planning refers to demand for family planning satisfied with modern methods of contraception. ***Condom use ta last sex. x refers that data was available and used to compute summary measures of respective indicator. DHS=Demographic and health survey; AIS=AIDS Indicator Survey; PHIA=Population-based HIV impact Assessment; MIS=Malaria Indicator Survey.

Melesse DY, *et al. BMJ Global Health* 2020; 5:e002231. doi: 10.1136/bmjgh-2019-002231

Supplement Table 2. Age at first sex, age at first marriage and age at first birth in years, HIV prevalence, and condom use at last sex, 33 countries, median value*, most recent survey since 2010

Indicator	Variable		Eastern & Southern Africa		West & Central Africa		Sub-Saharan Africa	
			Girls	Boys	Girls	Boys	Girls	Boys
First sex[1]	Number of countries		14	14	19	19	33	33
	Residence	Urban	18.8	18.5	17.6	18.8	18.2	18.6
		Rural	17.6	18.3	16.4	18.9	16.9	18.6
	Education	None / primary	16.9	18.2	16.2	18.9	16.7	18.8
		Secondary+	19.2	18.7	18.2	18.9	18.9	18.8
	Wealth quintile	Poorest (20%)	17.2	18.3	16.2	19.1	16.4	18.7
		Richest (20%)	19.6	18.6	18.2	18.8	18.6	18.7
First marriage[2]	Residence	Urban	21.7	26.9	21.0	27.6	21.3	27.2
		Rural	18.8	23.7	18.1	24.0	18.5	23.9
	Education	None / primary	18.7	22.9	18.1	24.1	18.2	23.8
		Secondary+	23.0	26.3	22.6	27.5	22.6	27.4
	Wealth quintile	Poorest (20%)	18.1	22.6	17.7	23.4	17.9	23.1
		Richest (20%)	23.0	26.8	22.6	28.1	22.8	27.3
First birth[1]	Residence	Urban	21.7	---	20.9	---	21.0	---
		Rural	19.5	---	18.9	---	19.1	---
	Education	None / primary	19.1	---	18.8	---	18.9	---
		Secondary+	22.4	---	22.7	---	22.5	---
	Wealth quintile	Poorest (20%)	19.1	---	18.7	---	18.8	---
		Richest (20%)	23.3	---	22.3	---	22.8	---
HIV prevalence[3]	Number of countries		12	12	16	16	28	28
	Residence	Urban	6.3	2.8	1.5	0.5	2.3	1.0
		Rural	4.5	2.3	0.9	0.4	1.1	0.5
	Education	None / primary	6.2	2.3	1.1	0.4	1.8	0.5
		Secondary+	4.3	2.4	1.2	0.4	1.6	0.7
	Wealth quintile	Poorest (20%)	4.1	2.3	0.6	0.5	1.0	0.6
		Richest (20%)	5.6	1.4	1.6	0.7	2.3	1.0
Condom use at last sex with non-regular partner[3]	Number of countries		14	14	18	18	32	32
	Residence	Urban	52.2	66.3	38.3	57.1	49.2	60.8
		Rural	34.3	47.4	25.2	40.3	27.1	43.4
	Education	None / primary	28.7	48.8	21.0	34.9	25.8	40.3
		Secondary+	50.1	57.7	45.5	54.2	47.6	55.7

Note: [1,2]Women are all age 25-29 years; [1]Men are all age 25-29 years; [2]Men are all age 25-29 years, and 30-34 if missing; [3]Women and men are all age 15-24 years. *Secondary+ = secondary or higher education level; None/primary refers to without or primary education; *Data extracted from STATcompiler (https://www.statcompiler.com/en/) and PHIA (only for HIV prevalence countries for few countries) (https://phia.icap.columbia.edu/).

B. Ten large-population countries analysis

The second component of the analysis involves the analysing of raw DHS and/or AIS data from the 10 large-population countries (Ethiopia, Ghana, Kenya, Malawi, Mozambique, Nigeria, Tanzania, Uganda, Zambia and Zimbabwe), with primary focus on adolescents of age 15-24 years. These countries have a total of over 15 million population and it is mainly to gain further insight into more recent trends, as well as to triangulate data and minimize the potential impact of recall bias on the key ASRH indicators.

A total of 29 national DHS and AIS data from 10 countries, including Ethiopia, Ghana, Kenya, Malawi, Mozambique, Nigeria, Tanzania, Uganda, Zambia and Zimbabwe, were used for analyses. Based on the median year of the first, second and third survey, we identified three common time points for the surveys, allowing a systematic assessment of trends: circa 2004, circa 2010 and circa 2015. All datasets with subnational focus were excluded from analysis. Below are the datasets utilized for this study.

Supplement Table 3: Surveys used for trend analysis by country

Country	Survey 1	Survey 2	Survey 3
Ethiopia	DHS 2005	DHS 2011	DHS 2016
Ghana	DHS 2003	DHS 2008	DHS 2014
Kenya	DHS 2003	DHS 2008-09	DHS 2014
Malawi	DHS 2004	DHS 2010	DHS 2015-16
Mozambique	DHS 2003	DHS 2011	AIS 2015
Nigeria	DHS 2003	DHS 2008	DHS 2013
Tanzania	DHS 2004-05	DHS 2010	DHS 2015-16
Uganda	DHS 2006	DHS 2011	DHS 2016
Zambia	DHS 2001-02	DHS 2007	DHS 2013-14
Zimbabwe	DHS 2005-06	DHS 2010-11	DHS 2015
Median year*	2004	2010	2015

DHS = Demographic and Health surveys; AIS = AIDS Indicator Survey; *Median year of data collection.

Descriptive analyses were conducted to describe the distribution of the proportion of adolescents attending school and use of contraceptives, while survival analysis was utilized to compute median age at first sex, marriage and birth. All analyses were done by survey for each country to examine trends over time. Our analyses to examine inequality was informed by our conceptual framework of looking at similarities and differences by cross-cutting variables: gender, urban vs. rural as residential area, current marital status, education status and socioeconomic status. Marital status was categorized as never married, currently married or formerly married (i.e., widowed, divorced or separated), while education status – categorized in three groups as no education, primary, or secondary or higher – was based on highest level of education completed during interview.

Survival analysis (*sts* function in the statistical package of Stata version 15) was performed to examine trends in the distribution of age at first sex, marriage and childbearing, whereas descriptive analysis was utilized to assess the level and trends in inequalities for education and sexual behaviour and health service utilization. The median survival time for age at first sex, marriage or childbirth was obtained from a cumulated single-year percent distribution from the Kaplan-Meier product-limit estimates of the survival curve, computed using a Stata function *stsum,* disaggregated by gender, urban-rural residential area, marital status, education status and socioeconomic status as appropriate. The Kaplan-Meier failure function was also utilized to explore the distribution of the probability of experiencing any of the first life events (i.e., first sex, marriage or childbirth) by each single year of age as appropriate. Analysis was performed by survey for each gender (male vs. female) and country.

The median age at first sex was determined based on whether or not the respondent ever had sex and, if applicable, recalled age at first sex. Reported age at first sex is set as the failure event and those who never had intercourse are censored at their current age during interview. We focused on data from all respondents 15-24 years and all analyses were conducted for each survey by sex of the respondent. The estimations of the distribution of age at first marriage and age at first childbirth were conducted in the same way as for age at first sex.

We focused on data from all respondents of age 15-24 years, except for median age at first marriage for men as the median was not reached by age 25 (analysis rerun using 15-29 years as reference). The median at first marriage for men was considerably later and not be computed for men 15-24 years for most countries. The analysis to estimate the distribution of median age at first birth was conducted only for adolescent women aged 15-24. The equity analyses involve triple disaggregation: age, sex and socioeconomic information, as well as several age-dependent indicators such as education status.

We also computed the median and weighted mean of the country values to describe the overall distribution of the key ASRH indicators and gain insight the big picture. The weighted mean was computed using data from UN country-year population estimates of respective age group adolescents. For each summary table, we provided country medians, and the weighted mean computed from country values.

Analysis of current school attendance among adolescent men and women aged 15-19 is based on data from all household members obtained through the household questionnaires. The percentage distribution of school attendance was computed by using the number of

adolescents who are currently attending or attended sometime during the current school year as numerator divided by the total number of adolescents in the respective age group.

The demand for family planning satisfying by modern methods among adolescent and young women aged 15-24 was computed as current contraceptive use divided by the sum of current use and unmet need for family planning, while modern contraceptive use among those who are sexually active was computed as the number of sexually active adolescent and young women using any modern contraceptive method (implants, IUD, injectable, pills, emergency contraception, condom, sterilization, other) as the numerator divided by the total number of sexually active adolescent and young women with a need of family planning (the denominator). Methods prevalence was computed by dividing the number of adolescent and young women using a particular method by total number of adolescent and young women who mentioned using any modern contraceptive method. Methods with less than 1% were grouped together in the 'others' category.

In this analysis we did not opt to show confidence intervals. For the median ages at specific events as it would imply having to introduce another measure such as the proportion who had experienced an event by age x. In this paper we look at the big picture and if the majority of the 10 countries are more or less uniformly moving in a certain direction, we make note of this in the main text. However, the confidence intervals of proportions from the life table analyses on sex, marriage and birth, and the trends in contraception, condom use and HIV have been computed and are available upon request.

Supplement Table 4. Percent of boys and girls 15-19 years who are attending school, DHS and AIS surveys

	Girls			Boys		
**Country**	**2004**	**2010**	**2015**	**2004**	**2010**	**2015**
Ethiopia	44.7	55.9	54.1	57.4	58.2	60.5
Ghana	47.9	53.4	45.4	60.4	62.7	52.7
Kenya	51.6	70.5	72.8	65.3	81.6	79.4
Malawi	46.4	56.4	54.2	64.7	67.9	71.7
Mozambique	41.4	42	37.2	61.8	66.4	53.4
Nigeria	47.9	54.2	48.1	67.1	68	61.7
Tanzania	26.4	39.2	34.5	42.6	49.5	39.6
Uganda	53.9	58.1	51.9	63.8	72.5	60.3
Zambia	38.3	55	55.8	58.6	77.5	68.3
Zimbabwe	42.2	46.3	53.4	54.2	56.4	60.8
**Median**	**45.5**	**54.6**	**52.6**	**61.1**	**67.1**	**60.7**
**Mean*(weighted)**	**43.1**	**51.5**	**52.0**	**61.0**	**65.4**	**60.5**

Note: Women and men are all 15-29 years. *Weighted by county-year population size of respective age group adolescents using data obtained from UN population database (https://population.un.org/wpp/). DHS=Demographic and Health survey; AIS=AIDS Indicator Survey. c., which stands for circa, refers to median years of surveys 1, 2 and 3.

Supplement Table 5. Age at first sex in years, median value, most recent surveys with median year of survey 2015

Country	Sex	All	Residential area		Education level			Wealth tercile		
			Urban	Rural	None	Primary	Secondary+	Lower	Middle	Higher
Ethiopia	Female	19.2	21.5	18.6	16.7	18.9	22.7	17.9	21.0	18.9
	Male	22.9	22.9	22.9	21.9	22.9	23.4	22.5	22.9	23.4
Ghana	Female	18.1	18.6	17.6	17.2	17.0	18.5	17.3	17.9	19.1
	Male	19.4	19.4	19.3	20.9	19.4	19.4	20.1	18.9	19.4
Kenya	Female	18.3	18.7	18.0	16.2	17.1	19.1	17.4	18.2	19.0
	Male	17.8	17.8	17.8	18.4	17.5	18.0	17.6	17.7	18.1
Malawi	Female	17.4	18.0	17.3	15.9	16.9	18.6	16.9	17.3	18.1
	Male	17.5	18.6	17.2	16.7	17.0	18.2	16.9	17.3	18.2
Mozambique	Female	16.2	16.8	15.9	15.5	15.9	16.9	15.8	15.9	16.8
	Male	16.5	16.5	16.6	16.5	16.2	16.8	15.9	16.7	16.7
Nigeria	Female	17.8	19.1	16.7	15.6	16.8	19.2	15.9	18.1	19.4
	Male	20.9	21.5	20.7	22.1	20.9	20.8	21.8	20.8	20.7
Tanzania	Female	17.4	18.0	17.0	15.7	16.8	18.8	16.5	17.3	18.5
	Male	17.9	18.1	17.7	17.9	17.3	18.6	17.4	17.8	18.0
Uganda	Female	17.6	18.3	17.4	16.8	17.0	18.4	17.0	17.6	18.3
	Male	17.9	17.8	18.0	18.4	17.7	18.2	18.1	17.7	18.0
Zambia	Female	17.5	18.2	16.8	16.3	16.6	18.1	16.6	17.1	18.7
	Male	17.9	18.6	17.0	16.3	17.2	18.3	16.9	17.2	19.1
Zimbabwe	Female	18.6	19.8	18.0	15.5	16.9	19.2	17.6	18.4	20.4
	Male	19.5	19.8	19.4	19.2	18.8	19.8	19.3	19.2	20.0
**Median**	**Female**	**17.7**	**18.6**	**18.0**	**18.4**	**17.7**	**18.6**	**18.1**	**17.8**	**19.1**
	**Male**	**17.9**	**18.3**	**17.3**	**15.9**	**16.9**	**18.6**	**16.9**	**17.6**	**18.7**
**Mean* (weighted)**	**Female**	**18.0**	**19.2**	**17.3**	**16.1**	**17.2**	**19.6**	**16.8**	**18.4**	**18.9**
	**Male**	**19.7**	**19.9**	**19.6**	**19.6**	**19.3**	**20.0**	**19.5**	**19.5**	**20.0**

Note: Women are all 15-24 years; Men 15-24 were used to estimate age first sex; *Weighted by county-year population size of respective age group adolescents using data obtained from UN population database (https://population.un.org/wpp/).

Supplement Table 6: Age at first marriage in years, median value, most recent surveys with median year of survey 2015.

Country	Sex	All	Residential area		Education level			Wealth tercile		
			Urban	Rural	None	Primary	Secondary+	Lower	Middle	Higher
Ethiopia	Female	19.8	23.4	18.8	17.0	19.4	23.7	17.7	18.7	22.0
	Male	25.3	27.2	24.3	22.6	24.1	27.4	23.8	24.6	26.9
Ghana	Female	23.5	25.0	23.2	18.6	20.8	25.0	20.3	23.8	25.8
	Male	28.1	≥30	25.9	27.4	25.5	28.7	25.7	27.7	≥30
Kenya	Female	21.2	21.7	20.9	17.3	19.4	22.9	19.2	21.0	22.5
	Male	25.7	26.2	25.4	23.3	24.4	26.8	24.2	26.1	26.6
Malawi	Female	18.9	21.2	18.6	17.3	18.1	21.5	17.9	18.5	20.7
	Male	23.3	25.9	22.7	22.0	21.8	24.7	21.7	22.9	25.5
Mozambique	Female	18.9	19.3	17.4	16.5	17.1	20.5	16.9	17.3	19.9
	Male	21.9	24.0	21.6	20.3	20.5	22.7	20.1	21.3	24.5
Nigeria	Female	18.1	22.9	17.5	15.7	17.8	23.6	15.9	19.7	23.7
	Male	28.3	≥30	25.7	23.2	26.3	≥30	24.0	29.1	≥30
Tanzania	Female	19.7	21.8	18.8	16.9	18.6	22.8	18.1	19.4	22.5
	Male	24.6	25.5	23.8	23.5	25.7	21.6	22.8	24.7	25.7
Uganda	Female	19.6	21.1	18.9	17.8	18.4	21.5	18.2	19.2	21.4
	Male	23.6	25.5	22.9	24.9	22.2	23.0	22.1	23.1	25.8
Zambia	Female	20.4	22.8	19.0	17.4	18.2	22.6	18.3	19.4	22.7
	Male	24.9	27.1	23.3	22.7	23.0	26.2	22.8	24.5	28.5
Zimbabwe	Female	19.6	21.8	18.7	16.6	17.5	20.4	18.2	19.2	22.1
	Male	25.1	26.8	23.9	26.4	23.7	25.4	23.7	24.4	27.1
**Median**	**Female**	**19.7**	**21.8**	**18.8**	**17.1**	**18.3**	**22.7**	**18.1**	**19.3**	**22.3**
	**Male**	**25.0**	**26.1**	**23.9**	**23.2**	**23.9**	**25.4**	**23.3**	**24.5**	**26.2**
**Mean***	**Female**	**19.7**	**21.8**	**18.8**	**17.1**	**18.3**	**22.7**	**18.1**	**19.3**	**22.3**
**(weighted)**	**Male**	**25.1**	**26.1**	**23.9**	**23.2**	**23.9**	**25.4**	**23.3**	**24.5**	**26.2**

Note: Women are all 15-24 years; Men 15-29 were used to estimate age first marriage; *Weighted by county-year population size of respective age group adolescents using data obtained from UN population database (https://population.un.org/wpp/).

Supplement Table 7: Age at first birth in years among women aged 15-24, median value, 9 countries (excluding Mozambique), most recent surveys (c.2015).

Country	All	Residence area		Education level			Wealth tercile		
		**Urban**	**Rural**	**None**	**Primary**	**Secondary+**	**Lower**	**Middle**	**Higher**
Ethiopia	21.9	**≥25**	20.7	19.0	21.5	**≥25**	19.9	21.3	24.7
Ghana	23.1	**≥25**	21.0	19.7	19.7	24.8	20.7	22.9	22.7
Kenya	20.9	21.8	20.3	18.7	19.1	22.7	19.5	20.8	20.9
Malawi	19.4	20.9	19.1	18.0	19.1	23.8	18.8	19.2	20.9
Mozambique	***	***	***	***	***	***	***	***	***
Nigeria	20.7	23.4	19.3	17.9	19.2	23.8	18.4	20.9	24.3
Tanzania	19.9	21.6	19.3	18.2	19.0	22.5	18.8	19.7	22.5
Uganda	19.8	21.2	19.4	18.3	18.9	21.5	18.9	19.7	21.4
Zambia	19.4	20.6	18.7	18.1	18.3	20.5	18.5	19.0	22.2
Zimbabwe	20.1	22.1	19.3	17.4	18.5	20.8	18.9	19.5	22.7
**Median**	**20.1**	**21.8**	**19.3**	**18.2**	**19.1**	**22.7**	**18.9**	**19.7**	**22.5**
**Mean*(weighted)**	**19.8**	**22.5**	**18.8**	**17.4**	**18.6**	**22.7**	**18.1**	**19.6**	**22.5**

Note: ***No data on age of first birth was available for Mozambique in survey c.2015; Women are all 15-24 years; *Mean weighted by county-year population size of adolescents of respective age group and survey year (population size data obtained from UN population database (https://population.un.org/wpp/)); In order to just compute the weighted mean, women 15-29 from Ethiopia and Ghana were used to estimate the median age at first birth because median age was found to be ≥25.

Supplement Table 8. Age at first sex, age at first marriage and age at first birth in years, weighted mean value#, using survey data since 2000.

			Females			Males			Male - female gap***		
			2004	2010	2015	2004	2010	2015	2004	2010	2015
Age at first sex		All	17.8	18.1	18	19.6	19.9	19.7	1.8	1.8	1.7
	Residence	Rural	17.3	17.5	17.3	19.5	19.7	19.6	2.2	2.2	2.3
		Urban	19.0	19.1	19.2	19.7	20.1	19.9	0.7	1.0	0.7
		Gap	1.7	1.6	1.9	0.2	0.4	0.3			
	Education level	None	16.2	16.2	16.1	19.9	19.6	19.6	3.7	3.4	3.5
		Primary	17.8	17.6	17.2	19.1	19.8	19.3	1.3	2.2	2.1
		Secondary+	19.8	19.6	19.6	19.9	20.1	20	0.1	0.5	0.4
		Gap*	3.6	3.4	3.5	0	0.5	0.4			
	Wealth tertile	Poorest	16.8	16.9	16.8	18.6	19.6	19.5	1.8	2.7	2.7
		Middle	18	18.3	18.4	14.4	19.9	19.5	-3.6	1.6	1.1
		Wealthiest	18.4	18.9	18.9	18.8	20.1	20	0.4	1.2	1.1
		Gap**	1.6	2	2.1	0.2	0.5	0.5			
Age at first marriage		All	18.9	19.2	19.5	24.8	25.1	25.3	5.9	5.9	5.8
	Residence	Rural	18.5	18.9	18.7	24.1	24.1	24.1	5.6	5.2	5.4
		Urban	17.7	21.3	22.5	19.2	26.7	23.1	1.5	5.4	0.6
		Gap	-0.8	2.4	3.7	-4.9	2.7	-1			
	Education level	None	16.6	16.7	16.7	25.5	23.7	24.3	8.9	7.0	7.6
		Primary	19.3	18.9	18.5	19.5	24.4	24.1	0.2	5.5	5.6
		Secondary+	24.8	24.5	23	25.7	26.2	25.7	0.9	1.7	2.7
		Gap*	8.2	7.9	6.3	0.2	2.5	1.4			
	Wealth tertile	Poorest	17.1	18.3	18.2	22.2	23.1	23.4	5.1	4.8	5.2
		Middle	17.7	19.0	19.5	23.1	24.6	24.6	5.4	5.6	5.1
		Wealthiest	20.0	21.5	22.3	25.2	26.6	26.6	5.2	5.1	4.3
		Gap**	2.9	3.2	4.1	2.9	3.5	3.2			
Age at first birth		All	20.4	20.8	19.8						
	Residence	Rural	19.7	19.9	18.8						
		Urban	23.4	23.3	22.5						
		Gap	3.7	3.4	3.8						
	Education level	None	18.5	18.3	17.4						
		Primary	19.9	19.6	18.6						
		Secondary+	24.0	24.3	22.7						
		Gap*	5.5	6.0	5.3						
	Wealth tertile	Poorest	18.2	19.0	18.1						
		Middle	19.0	20.5	19.6						
		Wealthiest	21.9	23.4	22.5						
		Gap**	3.6	4.4	4.4						

Note: Women are all 15-24 years; Men 15-24 were used to estimate age first sex; Men 15-29 were used to estimate age first marriage. c., which stands for circa, refers to median year of surveys of each country. *Absolute difference in median age between secondary+ (i.e., secondary or higher) and none (i.e., without education); **Absolute difference in median age between wealthiest and poorest wealth tercile; ***Absolute difference in median age between women and men; #Weighted by county-year population sizes of respective age group adolescents using data obtained from UN population database (https://population.un.org/wpp/).

Supplement Figure 1. Demand satisfied with modern family planning methods, women 15-24, median 9 countries (excluding Mozambique).

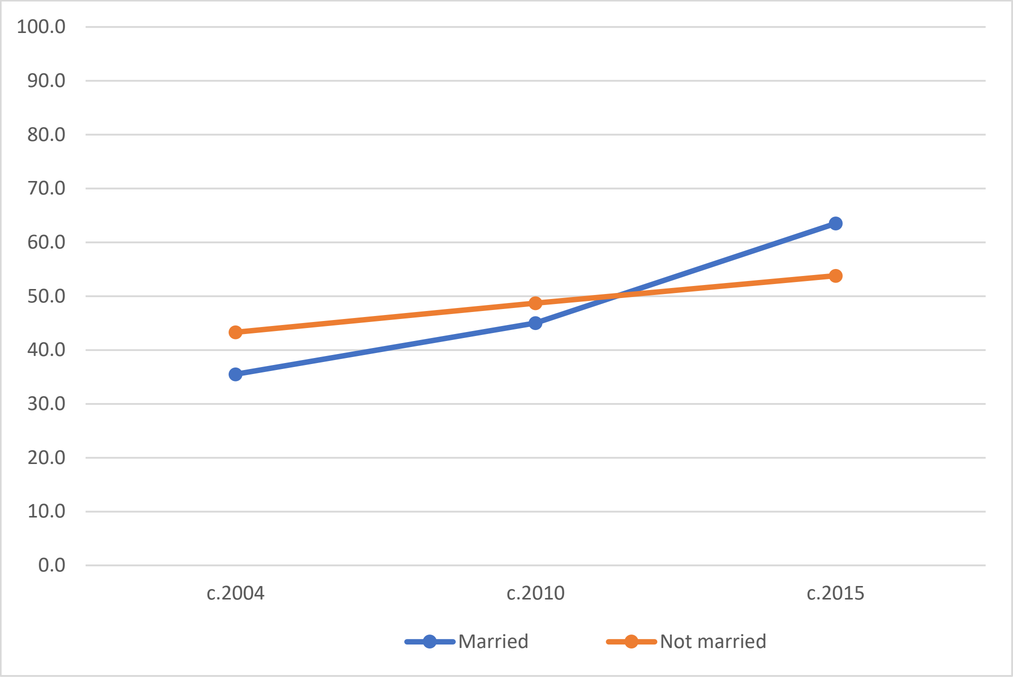

Supplement Figure 2. Demand for family planning satisfied with modern methods, women 15-24, by marital status, using DHS collected around 2004 and 2015.

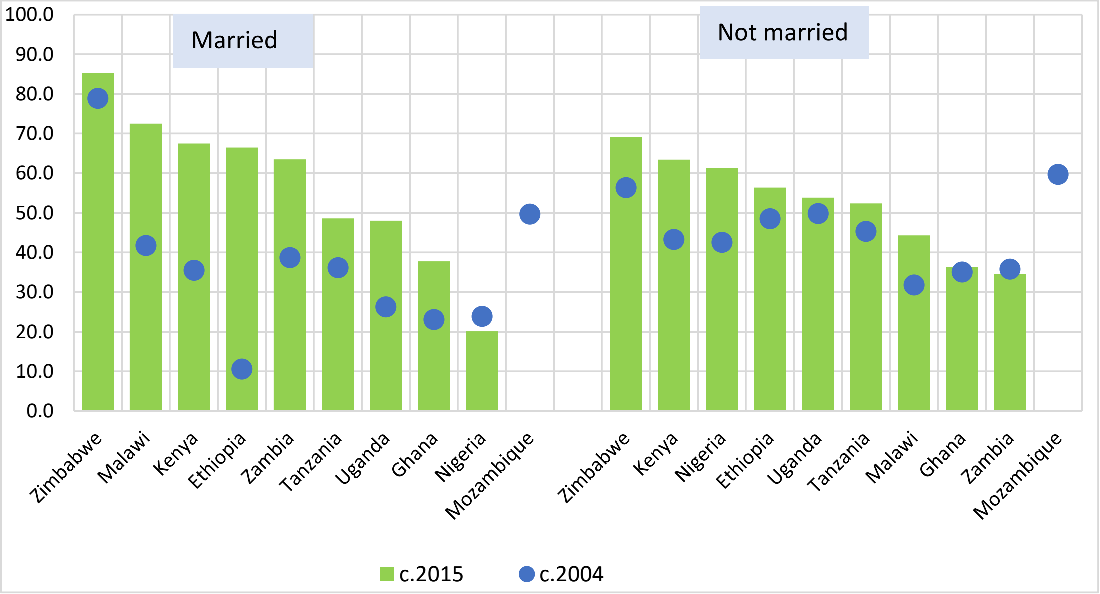

Melesse DY, *et al. BMJ Global Health* 2020; 5:e002231. doi: 10.1136/bmjgh-2019-002231

Supplement Figure 3. Demand satisfied for modern family planning methods by urban-rural residence, education and wealth quintile, women 15-24, country median, 10 countries, sub-Saharan Africa, using DHS collected around 2004 and 2015.

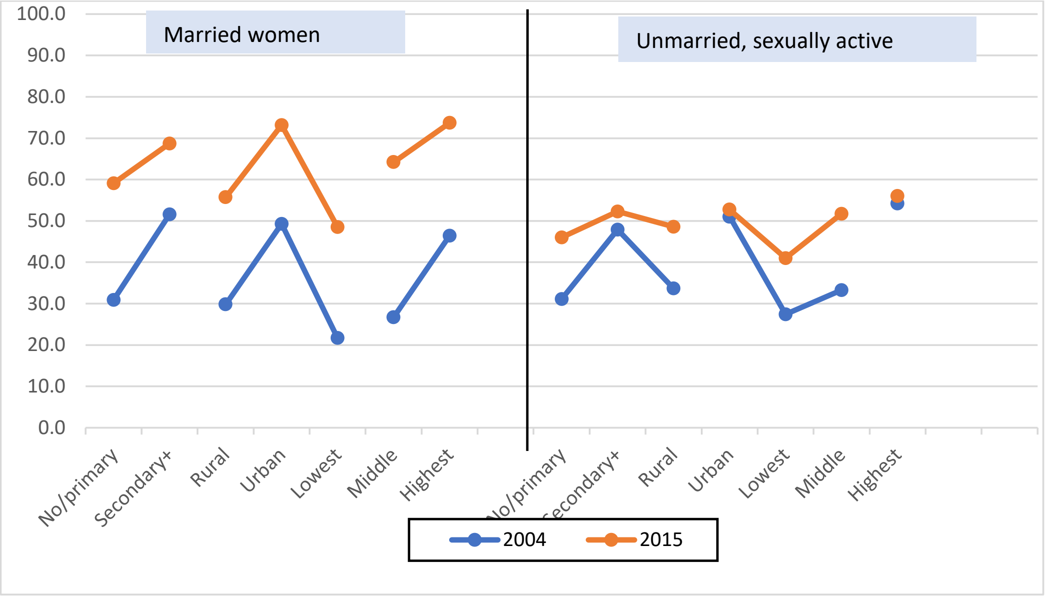

References

1 Demographic and Health Survey. Demographic and Health Surveys (various) [Datasets]. https://dhsprogram.com/data/ (accessed 20 Feb 2019).

2 ICAP at Columbia University. Population HIV impact assessment (PHIA) surveys project. 2019.https://phia.icap.columbia.edu/ (accessed 20 Jul 2019).

3 MacQuarrie KLD, Mallick L, Allen. Sexual and reproductive health in early and later adolescent: DHS data on youth age 10-19. DHS Comparative Reports No. 45: Rockville, Maryland, USA: ICF.: 2017. https://dhsprogram.com/pubs/pdf/CR45/CR45.pdf

4 ICF, 2012. The DHS Program STATcompiler. Funded by USAID. http://www.statcompiler.com (accessed 24 Sep 2018).
